# Steroid-Responsive Encephalopathy Associated With Autoimmune Thyroiditis Masquerading Sepsis

**DOI:** 10.7759/cureus.38826

**Published:** 2023-05-10

**Authors:** Jonathan Estaris, Surbhi Bansil, Yoshito Nishimura

**Affiliations:** 1 Medicine, University of Hawaii John A. Burns School of Medicine, Honolulu, USA

**Keywords:** altered mental state, antithyroid antibodies, psychosis, encephalopathy, autoimmune thyroiditis

## Abstract

A 72-year-old male presented with a fever and altered mental status. While initially diagnosed with sepsis due to cholangitis, he continued to decline and had seizures that complicated the course. After extensive workup, he was found to have anti-thyroid peroxidase antibodies and diagnosed with steroid-responsive encephalopathy associated with autoimmune thyroiditis (SREAT). He showed remarkable improvement with glucocorticoids and intravenous immunoglobulins. SREAT is a rare autoimmune encephalopathy characterized by elevated serum titers of antithyroid antibodies. SREAT needs to be listed as a differential diagnosis in a patient with encephalopathy of unclear causes, and the presence of antithyroid antibodies is a hallmark of the entity.

## Introduction

Steroid-responsive encephalopathy associated with autoimmune thyroiditis (SREAT) or Hashimoto encephalopathy is a rare autoimmune encephalopathy characterized by the presence of elevated serum titers of anti-thyroid peroxidase (anti-TPO) and/or anti-thyroglobulin (anti-TG) antibodies [1]. Although thyroid antibodies are present, patients are often clinically euthyroid, and evidence is lacking in support of a causal association between thyroid disease and encephalopathy [1,2]. SREAT may present with a spectrum of nonspecific neuropsychiatric signs and symptoms, including cognitive impairment and behavioral changes, altered consciousness, transient aphasia, psychosis, myoclonus, seizures, tremors, ataxia/gait disorders, sleep disturbances, or focal motor or sensory deficits, and often misdiagnosed as other conditions [1]. Given this considerable variation, a low threshold for recognition of SREAT is crucial. Here, we report a case of SREAT who initially presented with sepsis with altered consciousness, with a clinical course complicated by a new focal partial seizure.

## Case presentation

A 72-year-old male presented with a fever and altered mental status. His past medical history was significant for recurrent cholangitis associated with an obstructive ampullary adenoma, requiring recurrent endoscopic retrograde cholangiopancreatography (ERCP) and stent replacements. His family history and social history were noncontributory. The patient was initially found down on the ground of his home, altered and confused. He was brought to the Emergency Department, febrile at 38.1C, tachycardic at 106 beats per minute, hypertensive at 172/113 mmHg, and tachypneic at 28 respirations/minute. The patient was weak-appearing, unable to follow commands, and was not oriented to person, place, time, or situation. His responses to questions were slow, repetitive, and inappropriate. Initial laboratory tests were significant for a leukocytosis of 17,300 cells/μL, elevated alkaline phosphatase 329 IU/L, and elevated lactic acid of 2.4 mmol/L. Computed tomography of the head and abdomen were unremarkable. Our initial impression of his clinical picture was suggestive of sepsis secondary to acute cholangitis, and the patient was started on empiric antibiotics with Piperacillin/Tazobactam.

Neurologic examination was significant for gaze apraxia with a right-ward preference and left hemineglect. During the course, he developed a new focal partial seizure, with sudden onset of tonic contractions of his left arm, which gradually evolved retrogradely to involve his face. His acute seizure was managed appropriately, and he was started on maintenance anti-seizure prophylaxis with Phenytoin and Lacosamide. Antibiotic coverage was broadened for consideration of a suspected central nervous system infection, including herpes simplex encephalitis.

**Figure 1:**
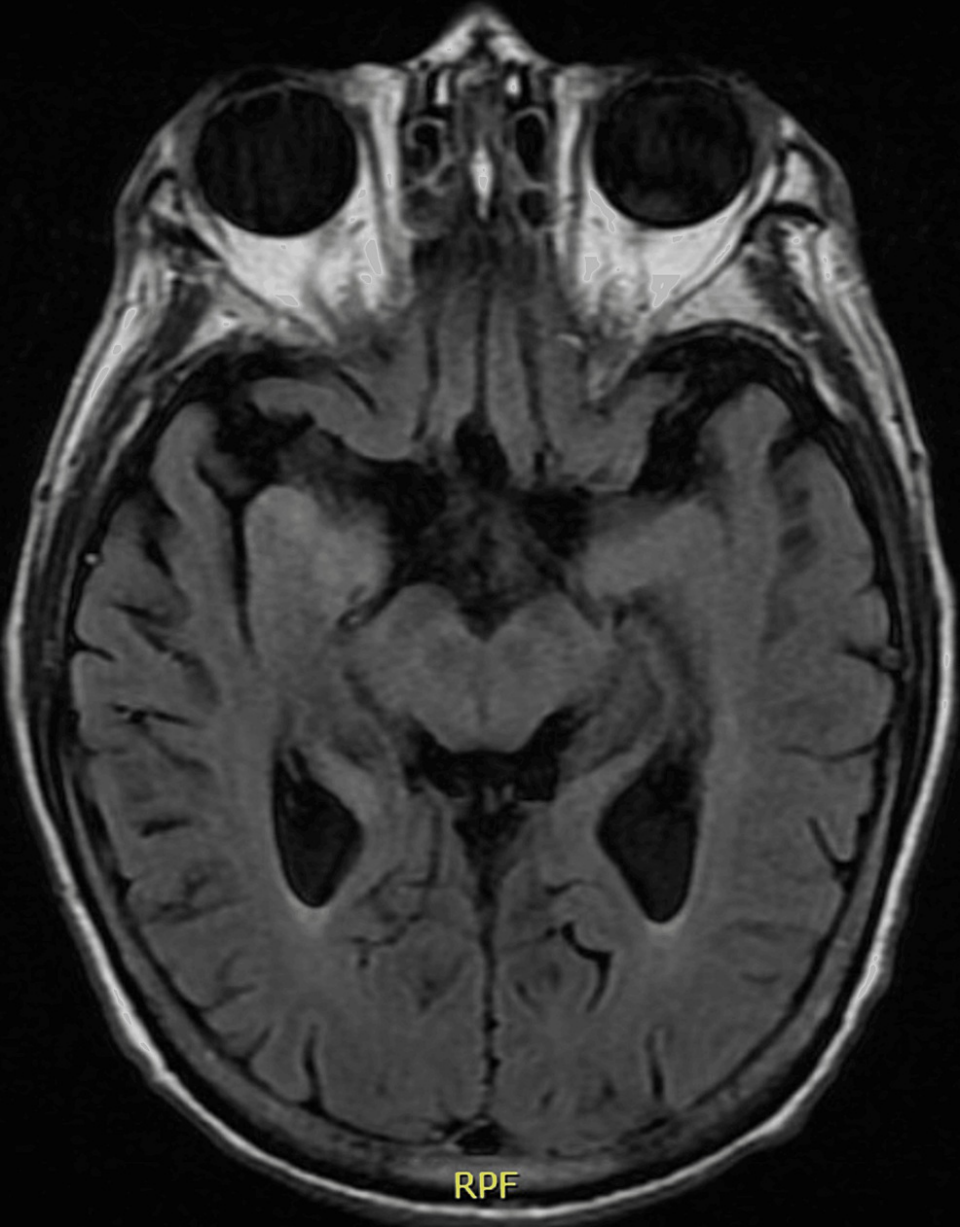
Brain magnetic resonance imaging Increased signal on fluid-attenuated inversion recovery imaging in the mesial right temporal lobe concerning right-sided limbic encephalitis.

His initial cerebrospinal fluid (CSF) analysis, sampled post-antibiotic administration, was significant for a pleocytosis, but a meningitis panel was unremarkable for an infectious etiology. Brain magnetic resonance imaging was suspicious for encephalitis with increased fluid-attenuated inversion recovery signal in the right temporal region (Figure 1).

An electroencephalogram confirmed an electrographic seizure with a right frontotemporal 1Hz spike and wave activity. At this point, suspicion was high for autoimmune encephalitis, as an infectious etiology was ruled out in the setting of the negative meningitis panel and cultures. Antibiotics were discontinued, and pulse dose methylprednisolone followed by high-dose prednisone and intravenous immunoglobulin (IVIG) was initiated pending a full workup for autoimmune encephalitis.

The patient showed a clinical response to the treatment and improved his mentation to his baseline. CSF anti-TPO antibodies were elevated at 39 IU/mL, while serum thyroid-stimulating hormone (TSH) was normal at 0.47 μIU/mL, and serum free T4 was 2.26 IU/mL. Additional ancillary workup for other potential etiologies of autoimmune encephalitis, including leucine-rich glioma-inactivated 1 antibodies, myelin oligodendrocyte glycoprotein antibodies, ganglioside GD1b IgG, and GM-1 IgG were negative. Given the results, the patient was diagnosed with SREAT.

## Discussion

This report summarized a case of autoimmune encephalitis secondary to SREAT initially presented as sepsis-causing encephalopathy. SREAT poses a diagnostic challenge as it may present a spectrum of varying neuropsychiatric clinical features, and it is often initially misdiagnosed as viral encephalitis or a neurodegenerative disorder [1,3]. In patients with SREAT, neuropsychiatric manifestations may occur independently of their thyroid status [1,2,4]. In a literature review of patients with SREAT, only 32% of patients had known overt thyroid disease, with the majority having clinical hypothyroidism with SREAT and a small minority having hyperthyroidism with Grave’s disease [4]. There were no uniform features or risk factors identified in those patients, but those with coma were more likely to be female (89% vs. 71%) and to have convulsions (84% vs. 40%). The median TSH was normal, but all patients had elevated serum titers of antithyroid antibodies, considered a hallmark of SREAT [4]. The majority of cases of SREAT have shown positive clinical responses to treatment with high doses of corticosteroids [5,6]. According to previous literature, 91% of patients showed partial or complete or complete responses with steadily declining levels of antithyroid antibodies following immunosuppressive therapy [4]. While the causation is still unclear, anti-TPO or anti-TG antibodies could be associated with disease activity in SREAT, and clinicians may need to follow up on the titers periodically.

The utility of steroid-sparing agents has been controversial. While optimal therapy is unknown, most patients were treated with intravenous methylprednisolone 500-1,000 mg/day for three to five days, followed by oral prednisone 1-2 mg/kg/day with a gradual taper based on the patient’s clinical status [6]. However, a small study showed the benefit of IVIG in cases complicated by significant neuropsychiatric symptoms such as seizures, as in our case [7,8].

## Conclusions

Clinicians should keep the diagnosis of SREAT on their differential for patients with ambiguous neuropsychiatric complaints such as prolonged coma or altered mental status of unknown causes after extensive workups to exclude common causes including drug intoxication, metabolic derangements such as hypoglycemia or electrolyte abnormalities, infection, uremia, and stroke. It is a potentially reversible cause of encephalopathy in patients, and the diagnosis should be considered even in those without overt thyroid disease. While it is a diagnosis of exclusion, the presence of antithyroid antibodies and a positive response to corticosteroid therapy support a diagnosis of SREAT.
